# Knowledge and Attitude towards Exclusive Breast Feeding among Mothers Attending Antenatal and Immunization Clinic at Dabat Health Center, Northwest Ethiopia: A Cross-Sectional Institution Based Study

**DOI:** 10.1155/2017/6561028

**Published:** 2017-09-18

**Authors:** Mulugeta Wassie Alamirew, Netsanet Habte Bayu, Nigusie Birhan Tebeje, Selam Fiseha Kassa

**Affiliations:** School of Nursing, University of Gondar College of Medicine and Health Sciences, P.O. Box 196, Gondar, Ethiopia

## Abstract

**Introduction:**

To assess knowledge and attitude towards exclusive breast feeding among mothers attending antenatal care and immunization clinic in Dabat Health Center, Northwest Ethiopia, 2016.

**Methodology:**

Institutional based descriptive cross-sectional study was conducted. The data was collected by using pretested, structured interview based questionnaires. The data were entered and analyzed using SPSS version 20.

**Result:**

A total of 384 participants were included in the study with a response rate of 100%. The majority were in the age groups of 20–30 (66.9%) and the mean age was 27.65; 325 (84.6%) were Orthodox Christianity followers. Majority were of Amhara ethnicity 370 (96.4%). Based on knowledge score, 268 (69.8%) were grouped as having good knowledge and regarding attitudinal score, 92 (24%) of the study participants were categorized as having negative attitude towards exclusive breast feeding (EBF) and the remaining 292 (76%) were categorized as having positive attitude.

**Conclusion:**

In this study, the knowledge of study participant mothers towards EBF is low which is less than three-fourths; however positive attitude towards EBF is more than three-fourths in this study. The authors recommend that health care workers who work in the areas of maternal and child health clinic should give appropriate information about EBF.

## 1. Introduction

Human milk is the ideal nourishment for infants' survival, growth, and development. Particularly in unhygienic conditions, however, breast milk substitutes carry a high risk of infection and can be fatal in infants [1]. Breast milk contains all the nutrients an infant needs in the first six months of life. Exclusive breast feeding means that the infant receives only breast milk [2].

Exclusive breast feeding in the first six months of life stimulates babies' immune systems and protects them from diarrhea and acute respiratory infections [1]. Exclusive breast feeding for the first six months of life is now considered as a global public heath goal that is linked to reduction of infant morbidity and mortality, especially in the developing world [3].

The world health organization (WHO) recommends exclusive breast feeding (EBF) for the first six months of life while it is advised to provide adequate and safe complementary foods with breast feeding for up to two years and beyond. EBF remains uncommon in most countries (both developed and developing), even in countries with high rates of breast feeding initiation. EBF rates in infants less than six months of age varied from as low as 20% in central and eastern European countries to 44% in south Asia [2, 4].

In Africa, more than 95% of infants are currently breastfed, but feeding practices are often inadequate; feeding water and other liquids to breastfed infants is a widespread practice [5]. The risk of morbidity is reduced by close to 70% when a child is exclusively breastfed. Exclusive breast feeding protected against serious morbidities in the first six months of life [6]. Research conducted at Ibadan, Nigeria, revealed that prevalence of mothers' knowledge towards EBF is still low, which accounts for about 36.2% and the same thing is true in Ethiopia, where it accounts for about 34.7% [7, 8]. Even though many researches are done about mother's knowledge and attitude towards exclusive breast feeding in many areas of Ethiopia, no research is done about it in and around Dabat Health Center. Due to this fact, this research is initiated and conducted, with the objective of assessing the knowledge and attitude towards exclusive breast feeding among mothers attending antenatal care and immunization clinic in Dabat Health Center, North Gondar zone, Northwest Ethiopia.

## 2. Methods

### 2.1. Study Design and Period

Institutional based descriptive cross-sectional study was conducted from March 10–30/2016 Gc.

### 2.2. Study Area

This study was conducted in Dabat Health Center. Dabat is found in northeast of Gondar 814 km far from Addis Ababa, the capital city of Ethiopia, and about 70 km from Gondar, the administrative town of North Gondar zone. It is about 2700 m above sea level. Currently, it has one health center and 3 health posts in the town plus 5 health posts around it. The current population is about 49,000.

### 2.3. Participants

Participants were all mothers visiting antenatal care and immunization clinic in Dabat Health Center during data collection.

### 2.4. Sample Size Determination and Sampling Technique

#### 2.4.1. Sample Size

We used single population proportion formula by taking proportion of knowledge of mothers towards exclusive breast feeding 65.1% from the previous research [9] and we used 95% confidence interval. To adjust nonresponse rate of study participants, we added 10% of the sample size.

(1) n=Za/22P1−pd2n=1.962  0.6511−0.6510.052=349+349∗10%=384,

where *a* is the level of significance which can be obtained as 1 − confidence interval, *P* is the prevalence of proportion of mothers' knowledge towards exclusive breast feeding which is equal to 65.1, *w* is the maximum acceptable difference (margin of error) which is equal to 5%, and *Za*/2 is the value under standard normal table for the given value of confidence level which is equal to 1.96.

### 2.5. Instruments

Data collection instruments consist of sociodemographic questionnaire (age, religion, ethnicity, marital status, educational status, occupation, residency, parity, gravidity, and ANC). To assess knowledge about exclusive breast feeding (EBF), nine knowledge questions were used (knowledge about exclusive breast feeding, the right time to give breast milk to a child after birth, what you do with the first milk or colostrum, right time to start complementary foods in addition to breast, foods and/or fluids recommended to give to a child under 6 months, if prelacteal feeding needed for an infant before starting breast milk, breast milk alone without water and other liquids being enough for an infant during the first 6 months of life, and exclusive breast feeding for the first 6 months being used to prevent diarrheal and respiratory diseases for the infant) and for assessment of attitude based on Likert scale (1 = strongly agree, 2 = agree, 3 = disagree, and 4 = strongly disagree) [4], attitude questionnaire was used. An average of responses on knowledge variables was done by computing variables and mothers who scored less than the average is labelled to have poor knowledge and those scored equal to or above the average score were considered as having good knowledge and all the attitude variables were computed and averaged. Those scored below the average were considered with negative attitude and those scored equal to or above the average were considered with positive attitude.

### 2.6. Data Collection Methods

A structured and pretested interviewer administrated questionnaire was used to gain data from study participants. First the questionnaire was translated to Amharic language which is a local language in understandable way to exclude misunderstanding to assure data quality. The questionnaire includes sociodemographic characteristics, knowledge, and attitude of study participants towards EBF.

### 2.7. Data Quality Assurance

Data quality was assured by using different approaches. First adequate orientation was provided for data collectors. After that, 5% of the questionnaires were pretested on volunteer mothers who have similar characteristics with study population in the same area who are not included in the study. After pretest, some questions were modified.

### 2.8. Data Processing and Analysis

After checking the completeness and appropriateness, the data were entered and coded into SPSS version 20.0 statistical package software for analysis. The result is presented in the form of frequencies and percentages by using tables, charts, and text.

### 2.9. Ethical Considerations

Ethical clearance was obtained from University of Gondar, College of Medicine and Health Sciences School of Nursing. All process was started after securing written permission from the School of Nursing Office. A permission letter obtained from School of Nursing was submitted to Dabat Health Center Office which is involved in the study. The study subjects were informed clearly and in detail about the importance of the study and a written consent was obtained. They have the right to refuse and withdraw from participating in the research without any explanation and they have the right to ask any question any time. In addition, the name of the study subjects was not included in the questionnaires which would address concern of the study subject.

## 3. Results

### 3.1. Sociodemographic Characteristics and Obstetrical History of Study Participants

A total of 384 mothers were interviewed successfully in this study which makes the response rate 100%. Majority of study participants were in the age groups of 20–30 (66.9%) and the mean age was 27.65, 325 (84.6%) were Orthodox Christianity followers, 56 (14.6%) were Muslims, and 3 (0.8%) were Protestants. Majority were Amhara ethnicity 370 (96.4%), 322 (83.9) were married, 33 (8.6%) were living together, 114 (29.7%) were not able to read and write, and 78 (29.7) completed secondary school. 173 (45.1%) were housewives and 82 (21.4%) were government employee. Majority 199 (51.8%) had four times ANC follow-ups. Eighty point five percent had 1–3 gravidity, and 67.7% had parity of 1–3. Majority (63.5%) of the respondents were urban dwellers while 36.5% lived in the rural areas (Table 1).

### 3.2. Knowledge of Study Participants towards Exclusive Breast Feeding

From the total study participants, based on knowledge score, 268 (69.8%) of the respondents were grouped as having good knowledge and 116 (30.2%) of the study participants were categorized as having poor knowledge. Three hundred fifteen (82%) knew about EBF and 69 (18%) do not know about EBF. Their major source of information was health institutions 255 (66.4%). Two hundred sixty-nine (70%) have good knowledge about right time to give breast milk to a child after birth.

One hundred seventy-four (45.3%) of the respondents have poor knowledge to give the first milk (colostrum) to the newborn, while 210 (54.7%) have good knowledge about it. The majority 250 (65.1%) knew that breast milk alone is enough for infants less than 6 months but 99 (25.8%) answered that breast milk alone is not enough for the infant under 6 months. Two hundred thirty-four (60.9%) of the participants knew that EBF prevents diarrheal and respiratory diseases as it is shown in Table 2.

### 3.3. Attitude of Study Participants towards Exclusive Breast Feeding

From this study based on the attitudinal score, 92 (24%) of the study participants were categorized as having negative attitude towards EBF and 292 (76%) were categorized as having positive attitude towards EBF. From study participants, 109 (28.4%) and 130 (33.9%) of them strongly disagree and disagree to the opinion discarding colostrum (first milk), respectively. For the opinion that starting complementary foods before 6 months is important, 12% strongly agree, 15.9% agree, 44% disagree and, 28.1% strongly disagree (Table 3).

## 4. Discussions

This study shows that mothers who have good knowledge towards EBF are 69.8% which is higher than a study conducted in Abha city, Saudi Arabia, which was 55.3% [10], and Nigeria that was 31% [11] and Guba Lafto woreda, Ethiopia (65.1%) [9]. Contrary to this, the finding is lower than a study conducted in Calabar, Nigeria, which was 80% [12] and Bedele town in Ethiopia where it is was 87.3% [13]. These differences may be due to variations in sampling technique, sociocultural status of study participants, health care delivery systems, and economic status of study participants.

In the current study, the knowledge of initiation of breast feeding is 70% which is less than that from a study conducted in Mizan Aman town of Ethiopia that was 73.3% [8] while it is much higher than that in a study conducted in Odisha which was 52.78% [14] as well as Tanzania which was 58.8% [15]. The above dissimilarities may be due to the differences of sample sizes and health care delivery systems.

Knowledge of respondents on colostrum in this study is 54.7% (having good knowledge). This is less than a study conducted in Mangalore which was 86.6% and in a study conducted in Mizan Aman town of Ethiopia that was 60.2%. This difference might be due to the difference of sample size.

In this study based on the attitudinal score, 76% of the respondents have positive attitude towards EBF, while 24% have negative attitude towards EBF. The finding is slightly greater than reports done in Rwanda, Kigali, which was 71.1% [16] and in Jima town in Ethiopia that was 73.9% [17]. However, the finding was less than a study result made in Nigeria, which is 84.7%, and Mizan Aman town, which is 89.5% in Ethiopia [8]. The disagreement among these study results might be attributed to these dissimilarities due to the differences of study settings.

In this study, 66.4% of study participants have information about EBF and their source of information was health institutions. This is more than the study conducted in Mizan Aman town, Southwestern Ethiopia, which was 62.7%, while it is much less than the study conducted in Kigali, Rwanda, that was 74.4 [8, 16]. And from this study, 29.4% mothers stated that babies before 6 months should receive BM and sugar. This is much less than the study conducted in Mvomero, Tanzania, which was 37.5% [15]. The difference may be due to methodological difference.

*Limitations of the Study*. Since it is difficult to generalize the result to the source population, this study shares the limitations of none probability sampling technique. This study also did not investigate the practice of exclusive breast feeding and it is only quantitative and not qualitative.

## 5. Conclusion

Knowledge of study participant mothers who attend ANC and immunization clinic towards exclusive breast feeding (EBF) is poor which is less than three-quarters. However, positive attitude towards EBF is more than three-fourths in this study. Based on this conclusion, health care workers who work in the areas of MCH clinic are better to give appropriate information about EBF to mothers who follow ANC and for those who come to immunization. In addition, health care providers who work in the areas of MCH should evaluate mothers' knowledge and attitude for every visit by asking questions related to EBF, health care officials of Dabat Health Center may play their role by actively following activities of health care providers of MCH, and health project implementers shall support and arrange training program for health care providers of MCH to improve mother's knowledge and attitude towards exclusive breast feeding.

## Figures and Tables

**Table 1:** Sociodemographic characteristics of the respondents in Dabat Health Center, Northwest Ethiopia, 2016. *n* = 384.

Characteristics	Frequency	Percent (%)
Ages of respondents		
<20	28	7.3
20–30	257	66.9
31–40	93	24.2
>40	6	1.6

Religion		
Orthodox	325	84.6
Muslim	56	14.6
Protestant	3	0.8

Ethnicity		
Amhara	370	96.4
Tigray	11	2.8
Agew	3	0.8

Marital status		
Married	322	83.9
Single	33	8.6
Divorced	19	4.9
Widowed	10	2.6

Educational status		
Not able to read and write	114	29.7
Can read and write (informal education)	58	15.1
Formal (1–8)	62	16.1
Secondary school (9-10)	78	20.3
10 + 2 and above	72	18.5

Occupational status		
Government employee	82	21.4
Private employee	15	3.9
Daily laborer	37	9.6
Housewife	173	45.1
Housemaid	12	3.1
Merchant	31	8.1
Farming	34	8.9

Residency		
Urban	244	63.5
Rural	140	36.5

Parity (1–3)	244	63.5

Gravidity (0–3)	309	80.5

ANC	199	51.8

**Table 2:** Knowledge of study participant mothers towards exclusive breast feeding, Dabat Health Center, Northwest Ethiopia, 2016. *n* = 384.

Variables	Frequency	Percent (%)
Do you know about exclusive breast feeding?		
Yes	315	82
No	69	18

Source of information		
Friends	10	2.6
Mass media	59	15.4
Health institution	255	66.4

Right time to give BM to a child after birth		
After giving some butter	31	8.1
Within an hour	269	70
After one hour	54	14.1
After 24 hours	30	7.8

What do you do with the first milk or colostrum?		
Discard	174	45.3
Feed immediately	210	54.7

Right time to start complementary foods		
3 months	10	2.6
4 months	34	8.8
5 months	23	6
6 months	311	81
7 months or above	6	1.6

Foods or fluids recommended to under 6 months' child		
Only breast milk	236	61.5
Breast milk and/or water or sugar	113	29.4
Infant formula	7	1.8
Others	28	7.3

Is prelacteal feeding needed?		
Yes	75	19.5
No	292	76.1
I do not know	17	4.4

BM alone is enough for infant < 6 months of life		
Yes	250	65.1
No	99	25.8
I do not know	35	9.1

EBF prevents diarrheal and respiratory diseases		
Yes	234	60.9
No	62	16.2
I do not know	88	22.9

Knowledge score		
Good	268	69.8
Poor	116	30.2

**Table 3:** Attitude of study participant mothers towards exclusive breast feeding (EBF) in Dabat Health Center, Northwest Ethiopia. *n* = 384.

Variables	Frequency	Percent
Giving BM for a newborn immediately within an hour is important		
Strongly agree	48	12.5
Agree	63	16.4
Disagree	182	47.1
Strongly disagree	92	24

Discarding the first milk or colostrum is important		
Strongly agree	55	14.3
Agree	90	23.4
Disagree	130	33.9
Strongly disagree	109	28.4

Only BM may not be sufficient for 3 months' child		
Strongly agree	56	14.6
Agree	87	22.6
Disagree	150	39.1
Strongly disagree	91	23.7

Starting complementary foods before 6 months is important		
Strongly agree	46	12
Agree	61	15.9
Disagree	169	44
Strongly disagree	108	28.1

Attitudinal score		
Positive	292	76
Negative	92	24

**Table:** (a) Sociodemographic characteristics

Sr. number	Question	Response	Remark
(1)	Age	—————— years	

(2)	Religion	(1) Orthodox (2) Muslim (3) Catholic (4) Protestant (5) Other (specify) - - - - - - - - - -	

(3)	Ethnicity	(1) Amhara (2) Tigray (3) Afar (4) Agew (5) Other (specify) - - - - - - - - - -	

(4)	Marital status	(1) Married (2) Single (3) Divorced/separated (4) Widowed	

(5)	Educational status	(1) Not able to read and write (2) Can read and write or informal education (3) Primary (1–8) (4) Secondary school (9-10) (5) 10 + 2 and above	

(6)	Occupational status	(1) Government employee (2) Private employee (3) Daily-laborer (4) Housewife (5) Housemaid/servant (6) Merchant (7) Farmer (8) Others (specify) - - - - - - - - -	

(7)	Residency	(1) Urban (2) Rural	

(8)	Parity	- - - - - - - - - -	

(9)	Gravidity	- - - - - - - - - -	

(10)	ANC follow-up	(1) Only one time (2) Twice (3) Three times (4) Four times	

**Table:** (b) Questions about knowledge of mothers towards exclusive breast feeding

Sr. number	Question	Response	Remark
(1)	Do you know about exclusive breast feeding?	(1) Yes (2) No	

(2)	What is your source of information?	(1) Friends (2) Mass media (3) Health institutions	

(3)	Right time to give breast milk?	(1) After giving some butter (2) Within an hour (3) After one hour (4) After 24 hours	

(4)	What do you do with the first milk or colostrum?	(1) Discard (2) Feed immediately	

(5)	What is the right time to start complementary foods?	(1) 3 months or less (2) 4 months (3) 5 months (4) 6 months (5) 7 months or above	

(6)	What are the foods and/or fluids recommended to give a child under 6 months?	(1) Only breast milk (2) Breast milk and/or plain water (3) Infant formula food or animal milk? (4) Others (specify) …………	

(7)	Is prelacteal feeding needed for an infant before starting breast milk?	(1) Yes (2) No (3) I do not know	

(8)	Is breast milk alone being enough for an infant during the first 6 months of life?	(1) Yes (2) No (3) I do not know	

(9)	Is exclusive breast feeding for the first 6 months used to prevent diarrheal and respiratory diseases for the infant?	(1) Yes (2) No (3) I do not know	

**Table:** (c) Questions related to attitude of mothers towards exclusive breast feeding

Sr. number	Questions	Response	Remark
(1)	Giving breast milk for a newborn immediately within an hour after birth is important?	(1) Strongly agree (2) Agree (3) Disagree (4) Strongly disagree	

(2)	Discarding the first milk or colostrum is important before giving breast milk to a newborn?	(1) Strongly agree (2) Agree (3) Disagree (4) Strongly disagree	

(3)	Giving a child of three months only breast milk may not be sufficient and a child needs water and other fluids to prevent thirst?	(1) Strongly agree (2) Agree (3) Disagree (4) Strongly disagree	

(4)	Starting complementary foods to a child before six months is important?	(1) Strongly agree (2) Agree (3) Disagree (4) Strongly disagree	

## Appendix

### A. Information Sheet

A questionnaire prepared to assess Knowledge and Attitude towards Exclusive Breast Feeding among Mothers Attending Antenatal and Immunization Clinic at Dabat Health Center, North West Ethiopia, 2016.

*Introduction*. This information sheet and consent form is prepared to explain the study you are being asked to join. Please hear carefully and ask any questions about the study before you agree to join. Are wishing to participate, you need to understand and sign the consent form then you will be requested to give your response.

*Purpose*. The purpose of this research is to assess knowledge and Attitude towards Exclusive Breast Feeding among Mothers Attending Antenatal and Immunization Clinic at Dabat Health Center, North West Ethiopia. The study will be helpful in determining the current level of knowledge and attitude towards EBF.

*Procedure*. This study uses institutional based cross-sectional study design, through using interview based Questionnaire, mothers asked to respond to questions in voluntary bases.

*Risk and/or Discomfort*. There is no any risk or discomfort that the respondents will face by participating in this research except dedication of time for responding the questioner.

*Benefits*. If you participate in this research project, there may not be direct benefit to you but your participation is likely to help us in assessing the knowledge and your attitude towards EBF. Ultimately, this will help us to work on awareness creation on the benefits of EBF.

*Incentives/Payment*. There are no payments or incentives for respondents in this research.

*Confidentiality*. Any personal information registered in the questioner will not be copied and transferred to other bodies. All kinds of information will be kept confidential in a secured place.

*Right to Refuse*. You have full right to refuse or withdraw from participating in this research at any time you wish without losing any of your right.

### B. Consent Form

Hello! Good morning/good afternoon, my name is —————— and I am a data collector from university of Gondar, school of nursing. Thank you for allowing us to share your precious time and for your willingness to participate in this study. The objective of this study is to assess knowledge and attitude of mothers towards exclusive breast feeding, among Mothers Attending Antenatal and Immunization Clinic at Dabat Health Center, Northwest Ethiopia. You are chosen to participate in this study and the study will take about 10 minutes to complete the questionnaire.

We would greatly appreciate your help in responding to this survey.

Are you willing to participate?

  Yes
  No

Thank you for voluntarily participate in the study!

### C. Questionnaire

See Table 4.
